# Supplementary material for: Development of a Subunit Vaccine against Duck Hepatitis A Virus Serotype 3
Source: Vaccines (Basel). 2022 Mar 28;10(4):523. doi: 10.3390/vaccines10040523 (PMC9028120; doi:10.3390/vaccines10040523)
Supplement: Supplementary file 1 [file vaccines-10-00523-s001.zip › vaccines-1609252-supplementary.pdf]

Supplementary Materials

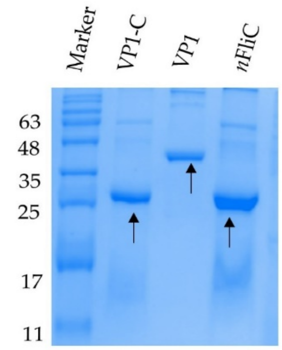

Figure S1. Full gel photo of SDS-PAGE of the recombinant proteins.

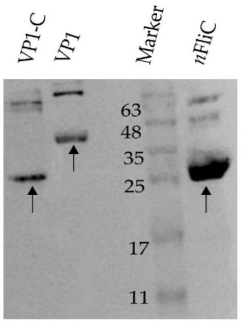

Figure S2. Full gel photo of Western blot analysis of the recombinant proteins.

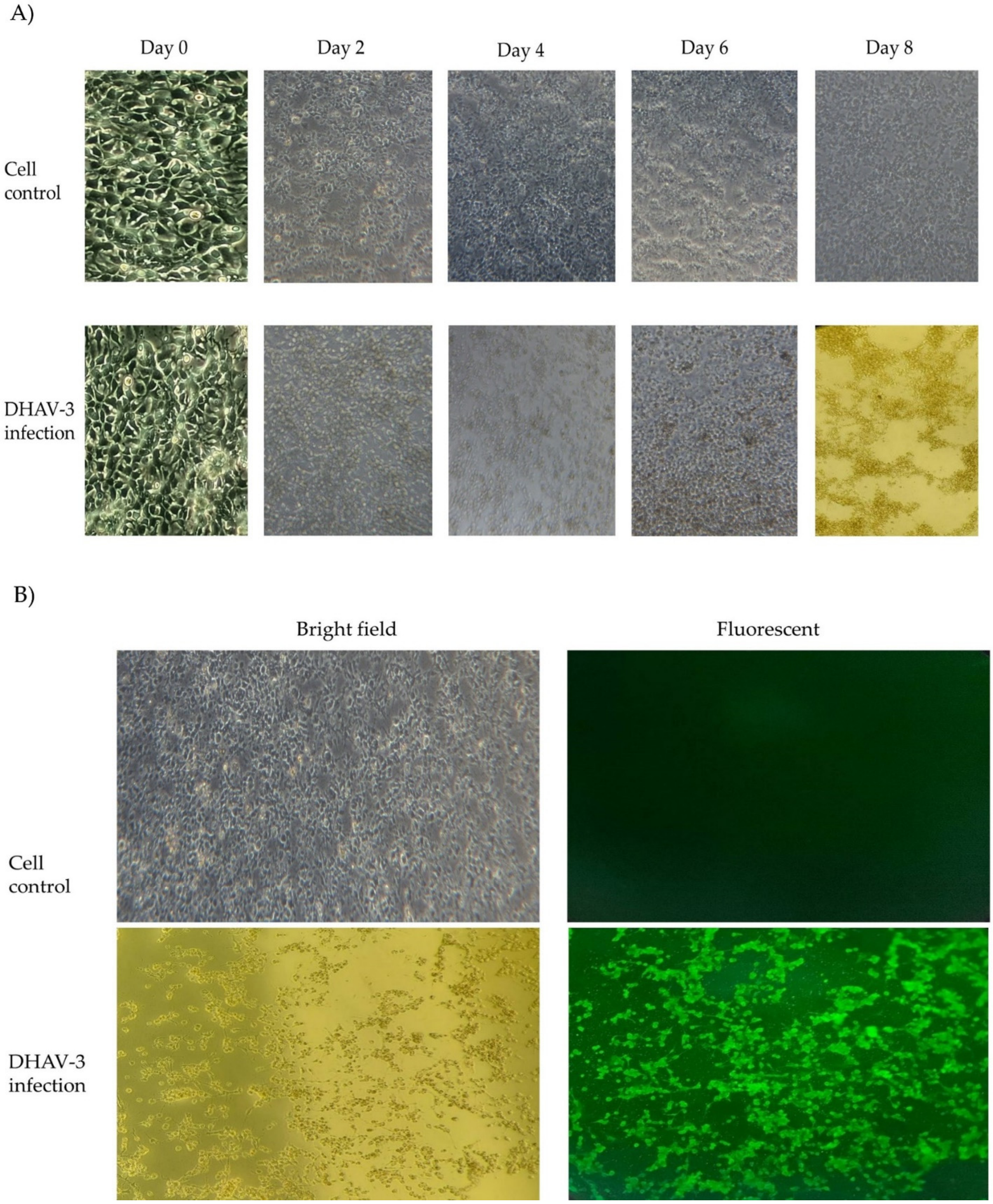

Figure S3. Cytopathic effect of DHAV-3 on the Leghorn Male Hepatoma cell line. (A) Cytopathic effect of DHAV-3 is shown after infection. (B) Indirect immunofluorescence assay of DHAV-3 infection. LMH cells were stained three day after infection with 100TCID$_{50}$ of DHAV-3. Primary antibody was from ducks vaccinated with VP1-C and KPL Fluorescein-labeled Antibody to Duck IgG (Seracare, Mildford, MA, USA) was used as secondary antibody.

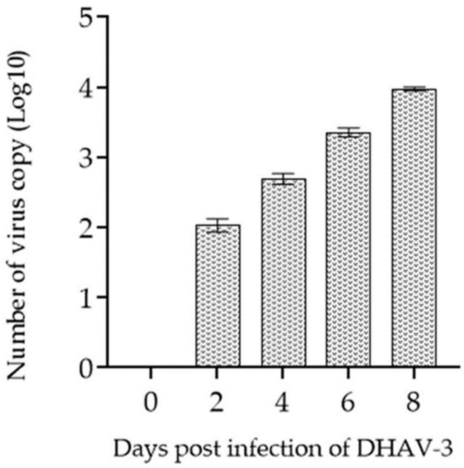

Figure S4. Viral copy numbers (log10) of DHAV-3 in the LMH confluent cell line as determined by RT-qPCR.

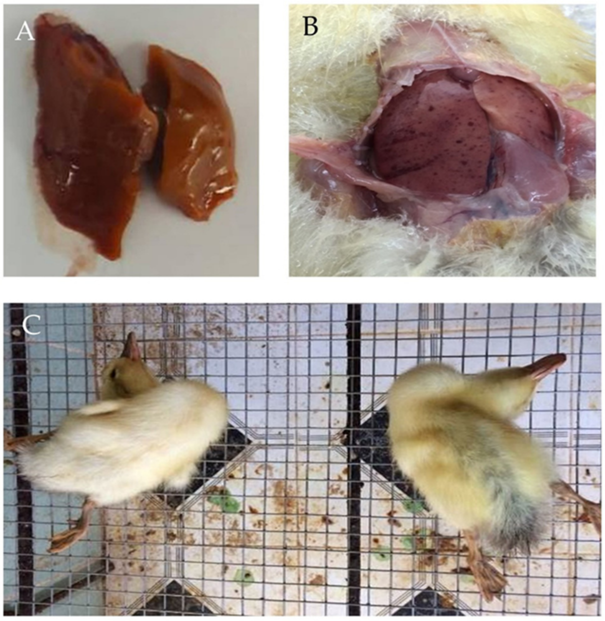

Figure S5. Gross lesions of ducklings infected with the DHAV-3 isolate, NT01. (A) Liver of uninfected duckling. (B) Liver of DHAV-3-infected duckling showing ecchymotic hemorrhage. (C) Opisthotonus posture of infected ducklings.

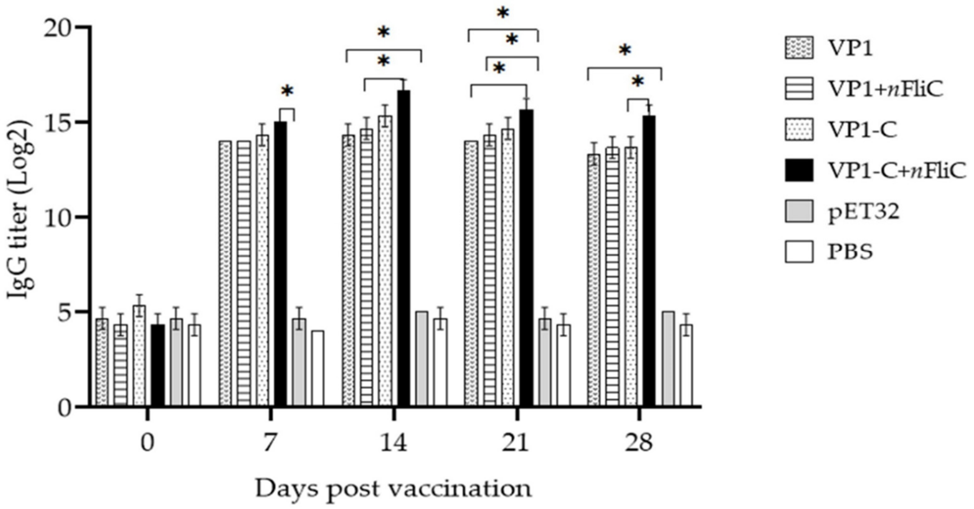

Figure S6. Total IgG titers of vaccinated ducklings against DHAV-3 were determined by indirect ELISA. An asterisk indicates statistically significant difference (p<0.05).
